# Warming Houses

**Published:** 1864-12

**Authors:** 


### WARMING HOUSES.

Of all the plans ever devised for heating our dwellings, that which combines in the highest degree the important qualities of healthfulness, chearfulness, and comfort, the Low-down Grate of T. S. Dixon, of Philadelphia, has no equal; and however the house may be heated in general, the family-room and the parlor of every man who consults the deepest enjoyment of his wife, children, and friends, will have the broad, blazing hearthful of glowing coals which Dixon's Grate alone affords. It burns wood, coke, soft and hard coal, as may be desired, giving out more heat without burning any more fuel than a common grate; giving no more dust than a common wood-fire, nor half as much; in real truth, not more than a furnace in the cellar sends through the register in our own house, not as much when properly managed.

The Maryland School Journal, published monthly at Hagerstown, Maryland, for one dollar a year, is edited with great judgment; every page is full of practical articles, calculated to instruct and make happy any family which follows them; every true lover of the noble old State of Maryland ought to order a copy for his household for 1865.

A Sermon, delivered by the Rev. Dwight K. Bartlett, at Hartford, Ct., in reference to the times, has been published at the urgent request of gentlemen in Albany, New-York. Mr. Bartlett is an Old-School Presbyterian clergyman, and although he has been preaching but two or three years, this Sermon shows that he is a rising man, and is destined to occupy a high position. He has already refused offers from some of the best pulpits of his native State of New-York.

